# Antibody-mediated enhancement aggravates chikungunya virus infection and disease severity

**DOI:** 10.1038/s41598-018-20305-4

**Published:** 2018-01-30

**Authors:** Fok-Moon Lum, Thérèse Couderc, Bing-Shao Chia, Ruo-Yan Ong, Zhisheng Her, Angela Chow, Yee-Sin Leo, Yiu-Wing Kam, Laurent Rénia, Marc Lecuit, Lisa F. P. Ng

**Affiliations:** 1 Singapore Immunology Network, Agency for Science, Technology and Research (A*STAR), Singapore, 138648 Singapore; 2 Department of Biochemistry, Yong Loo Lin School of Medicine, National University of Singapore (NUS), Singapore, 117596 Singapore; 3 Biology of Infection Unit, Institut Pasteur, Paris, 75015 France; 4 Inserm U1117, Paris, 75015 France; 5 Institute of Infectious Disease and Epidemiology, Tan Tock Seng Hospital, Singapore, 308433 Singapore; 6 Paris Descartes University, Sorbonne Paris Cité, Institut Imagine, Necker-Enfants Malades University Hospital, Division of Infectious Diseases and Tropical Medicine, APHP, Paris, 75743 France; 7 Institute of Infection and Global Health, University of Liverpool, Liverpool, L69 7BE UK; 8 Present Address: Department of Microbiology and Immunobiology, Harvard Medical School, Boston, Massachusetts 02115 USA; 9 Present Address: School of Life Sciences, University of Dundee, Dundee, DD1 5EH UK; 10 Present Address: Institute of Molecular and Cell Biology, Agency for Science, Technology and Research (A*STAR), Singapore, Singapore

## Abstract

The arthropod-transmitted chikungunya virus (CHIKV) causes a flu-like disease that is characterized by incapacitating arthralgia. The re-emergence of CHIKV and the continual risk of new epidemics have reignited research in CHIKV pathogenesis. Virus-specific antibodies have been shown to control virus clearance, but antibodies present at sub-neutralizing concentrations can also augment virus infection that exacerbates disease severity. To explore this occurrence, CHIKV infection was investigated in the presence of CHIKV-specific antibodies in both primary human cells and a murine macrophage cell line, RAW264.7. Enhanced attachment of CHIKV to the primary human monocytes and B cells was observed while increased viral replication was detected in RAW264.7 cells. Blocking of specific Fc receptors (FcγRs) led to the abrogation of these observations. Furthermore, experimental infection in adult mice showed that animals had higher viral RNA loads and endured more severe joint inflammation in the presence of sub-neutralizing concentrations of CHIKV-specific antibodies. In addition, CHIKV infection in 11 days old mice under enhancing condition resulted in higher muscles viral RNA load detected and death. These observations provide the first evidence of antibody-mediated enhancement in CHIKV infection and pathogenesis and could also be relevant for other important arboviruses such as Zika virus.

## Introduction

Chikungunya virus (CHIKV) is a member of the *Alphavirus* genus of the *Togaviridae* family^1,2^. It is responsible for chikungunya fever (CHIKF), a disease characterized by the presence of incapacitating arthralgia^3^. CHIKV is transmitted by arthropod vectors, such as the *Aedes aegypti* and *Aedes albopictus* mosquitoes, with the latter being implicated in the transmission of CHIKV during the 2005–2006 Indian Ocean outbreak and in Europe^4^. For the past decade, re-emergence of CHIKV has led to numerous outbreaks in different parts of the world: Asia^5–12^, Europe^4,13,14^ and islands in the Indian Ocean^15,16^. Outbreaks of CHIKV infections have also been reported in the Caribbean islands^17,18^ and CHIKV has since successfully invaded North, Central and South America^19^.

Enhancement of arbovirus infections via antibodies was first demonstrated in 1964^20^. This is a paradoxical phenomenon of antibodies forming complexes by binding to viruses, which then interact with cell surface receptors and promote entry into susceptible host cells, subsequently increasing virus replication^21,22^. This was observed for rabies virus^23^, influenza virus^24^, dengue virus (DENV)^25,26^, Ross River virus (RRV)^27^, human immunodeficiency virus (HIV)^28^ and Marburg virus^29^. Among alphaviruses, although virus enhancement was documented only in RRV infections^27,30–32^, most of these studies were conducted using *in vitro* murine cell line-based systems^27,31,32^. The development of a suitable infection system with primary human cells and an *in vivo* model allows the study of antibody enhancement in clinically important viruses, such as the recently emerged Zika virus (ZIKV), which infection is enhanced with cross-reactive anti-DENV antibodies^33^.

Here, we demonstrate antibody-mediated enhancement of CHIKV attachment and infection in primary human monocytes and B cells and a relevant murine cell line in the presence of sub-neutralizing levels of anti-CHIKV antibodies obtained from CHIKV-infected patients or animals. This enhancement was further demonstrated to mediate through the Fc receptors (FcγRs), with FcγRII being the key mediator. Importantly, two complementary animal models demonstrated enhanced CHIKV infections in the presence of sub-neutralizing levels of anti-CHIKV antibodies, with severe disease outcome and increase lethality. This study brings also caution to the importance of such undesired effects in anti-CHIKV vaccine designs.

## Results

### CHIKV-specific polyclonal antibodies mediate CHIKV infection enhancement in primary human cells

**Figure 1:**
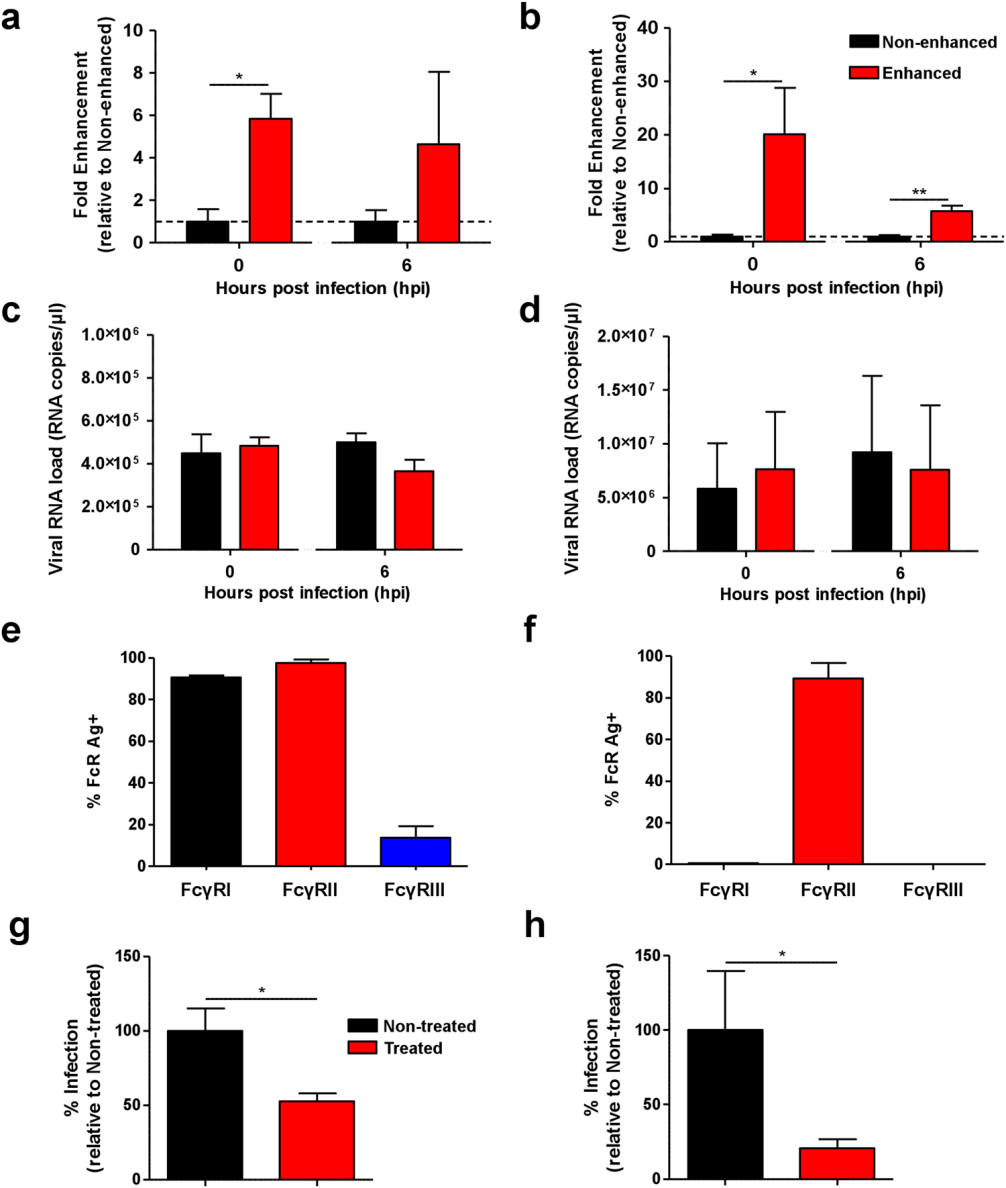
Enhancement of CHIKV infection in purified primary human cells. Primary human (**a**,**c**) monocytes and (**b**,**d**) B cells (2 × 10^6^ cells per infection) were infected with CHIKV (moi 10) in the presence (enhanced) or absence (non-enhanced) of diluted CHIKV-specific patient plasma containing total IgG at a concentration of 3.6 ± 2.9 μg/ml. (**a**,**b**) Detection of CHIKV antigen and (**c**,**d**) viral RNA load were determined at 0 and 6 hpi. Level of CHIKV antigen detected is expressed as fold enhancement relative to the non-enhanced infection controls. Data shown are mean ± SD from 3 independent experiments by parametric unpaired *t* test (**P = 0.0015 for 0 hpi monocytes; *P = 0.0458 for 0 hpi B cells and **P = 0.0053 for 6 hpi B cells). Expression of FcγRs in (**e**) monocytes and (**f**) B cells. Primary human (**g**) monocytes and (**h**) B cells (2 × 10^6^ cells per infection) were treated with 10 μg of FcγRs blocking agent prior to CHIKV infection (moi 10) under enhancing conditions (as above). Percentage of infection is expressed as level of CHIKV antigen detected relative to the non-treated infection controls at 0 hpi. Data are presented as mean ± SD from 4 independent experiments by Mann-Whitney *U* test (*P = 0.0286 for monocytes and *P = 0.0143 for B cells).

To investigate if sub-neutralizing concentrations of CHIKV-specific antibodies enhance CHIKV infection, diluted CHIKV-specific patients’ plasma obtained from a CHIKV cohort^8,34,35^ were mixed with CHIKV before being used to infect human primary monocytes and B cells. At low antibody concentration, antibody-mediated enhancement was shown to occur at antibody concentrations of 3.6 ± 2.9 μg/ml (Table 1). The presence of CHIKV antigen was detected by flow cytometry, where detection was increased by ~5 fold in monocytes (Fig. 1a) and by ~20 fold in B cells (Fig. 1b). However, active virus replication was not observed (Fig. 1c,d) in both cell types. Next, a Zs-Green tagged CHIKV variant was used for the infection of human whole blood. With this virus, a successful infection would lead to the production of the Zs-Green protein. Levels of infection can therefore be known through the detection of Zs-Green positive cells. It was observed that infection in the presence of patients’ plasma (total IgG concentrations of 1.8 ± 1.45 µg/ml) led to an increase in the numbers of Zs-Green positive monocytes. However, this was not observed in the B cells and plasmacytoid dendritic cells (pDCs) (Fig. S1a). Once again, the viral RNA load did not concur with enhanced infection (Fig. S1b).

**Table 1:** Quantification of total IgG in CHIKV-infected human patient plasma and mice sera.

Host	No. of samples	Sample collection	Mean concentration ± SD (mg/ml)^a^	Dilution leading to enhancement^b^
Human	9	2–3 months post illness onset	3.6 ± 2.9	1000–2000 folds
Mouse	5	15 days post infection	1.9 ± 0.47	100–10000 folds

^a^Concentration measured here refer to the total amount of IgG antibody present in the plasma and serum samples, including non-CHIKV-specific IgG antibodies.

^b^Dilutions listed here are derived from specific experiments conducted in this study.

### Enhancement in CHIKV infection is mediated by FcγRII

Fcγ receptors (FcγRs)-dependent pathway is the most common mechanism in antibody-mediated enhancement of infections^22^. Monocytes express FcγRI and FcγRII equally (Fig. 1e), while B cells express only FcγRII (Fig. 1f). To investigate if FcγRs play a role in antibody-mediated enhancement, cells were pre-incubated with 10 μg of FcγR blocking agent prior to CHIKV infection under enhancement. Blocking of FcγRs significantly reduced the level of CHIKV antigen detected in monocytes (Fig. 1g) and B cells (Fig. 1h).

**Figure 2:**
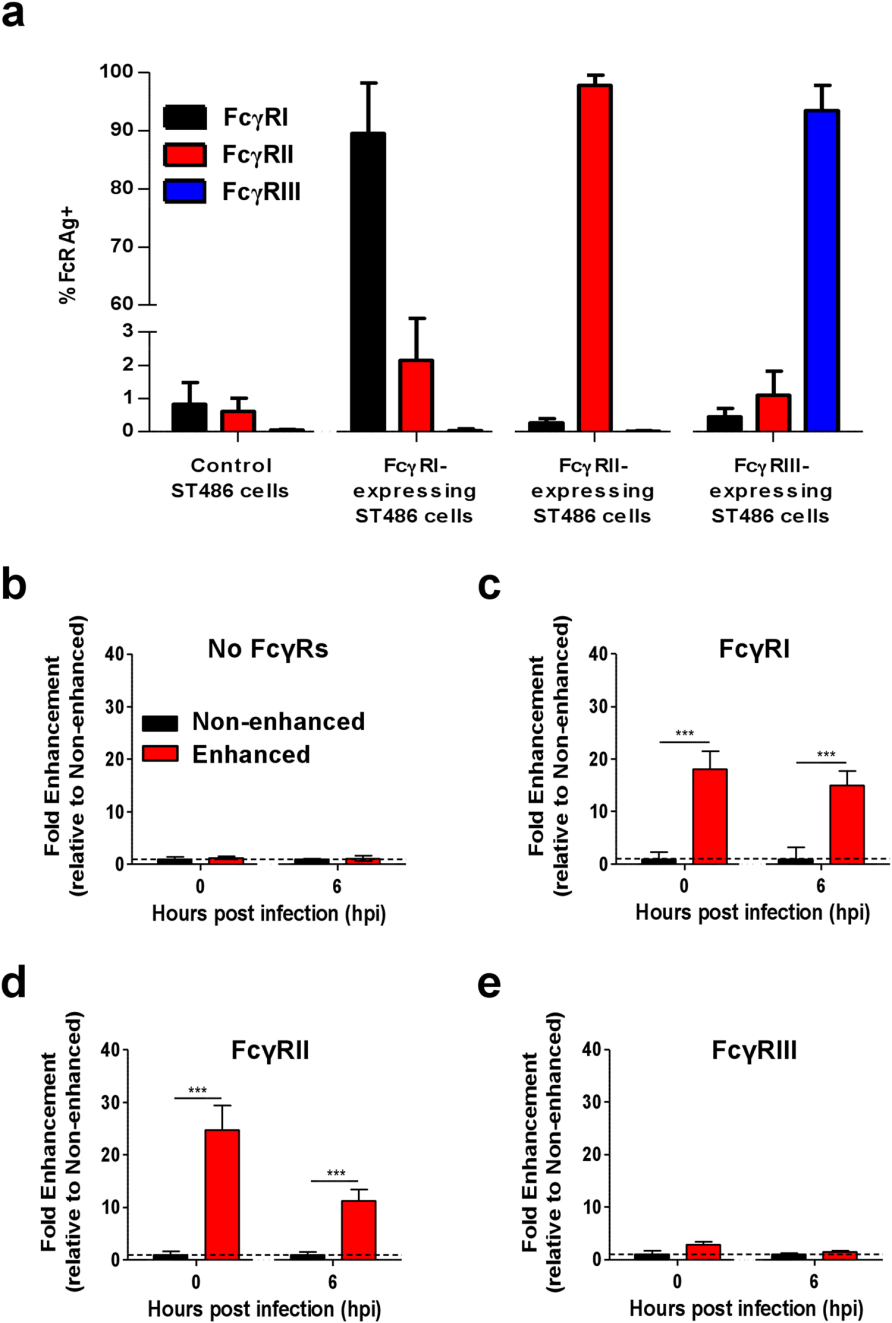
Enhancement of CHIKV infection is mediated via Fcγ receptors. (**a**) Expression of FcγRI, FcγRII and FcγRIII was determined in FcγR-expressing ST486 cell lines by flow cytometry (n = 3). CHIKV (moi 10) was used to infect (**b**) non-FcγR-expressing, (**c**) FcγRI-expressing, (**d**) FcγRII-expressing and (**e**) FcγRIII-expressing ST486 cell lines (2 × 10^6^ cells per infection) in the presence (enhanced) or absence (non-enhanced) of diluted patient plasma containing total IgG at a concentration of 3.6 ± 2.9 μg/ml (n = 9). Level of CHIKV antigen was determined at 0 and 6 hpi. Level of CHIKV antigen detected is expressed as fold enhancement relative to the non-enhanced infection controls. Data are presented as mean ± SD by Mann-Whitney *U* test (***P < 0.0001 for level of CHIKV infection observed at both 0 and 6 hpi in FcγRI and FcγRII expressing cells).

To elucidate which class of FcγRs enhances CHIKV infection, four different FcγR-expressing stable human B lymphocyte cell line, ST486, that have been modified to stably express a single class of FcγR driven by the same promoter^36^ were used (Fig. 2a). Parental ST486 cells naturally deficient in FcγRs expression were used as controls and were not susceptible to CHIKV infection (Fig. 2b). A significant increase in CHIKV antigen was detected in ST486 cells over-expressing FcγRI or FcγRII when infection was performed in the presence of low levels of CHIKV-specific antibodies (Fig. 2c,d). ST486 cells over-expressing FcγRIII neither supported nor enhanced CHIKV infection (Fig. 2e).

### Antibody-mediated enhancement of CHIKV infection in human macrophages

**Figure 3:**
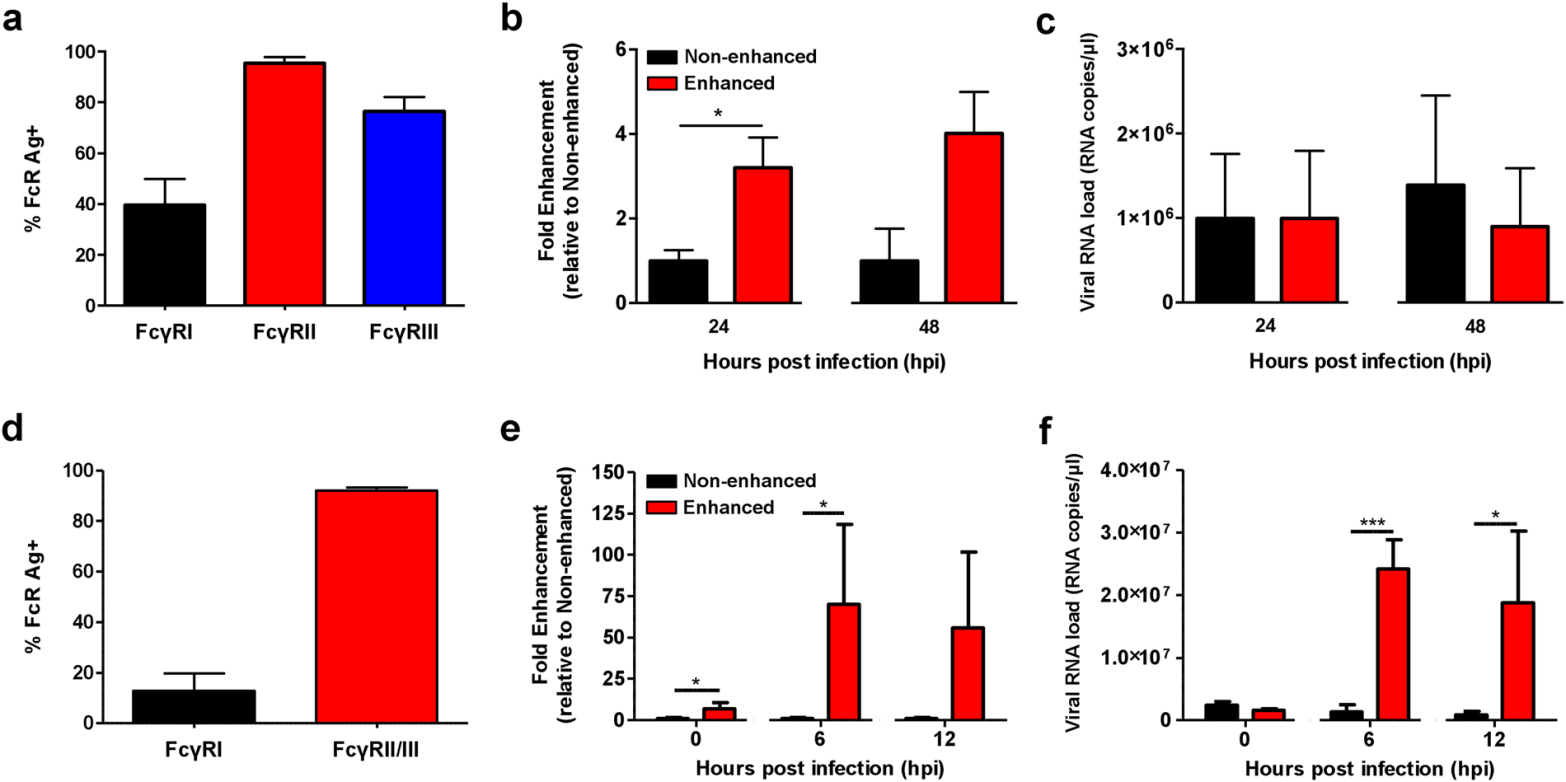
CHIKV infection enhancement in macrophages from humans and mice. (**a**) Expression of FcγRs in human monocytes-derived macrophages (MDMs) was detected with flow cytometry. Human MDMs (2 × 10^6^ cells per infection) were infected with Zs-Green tagged CHIKV (moi 10) either in the presence (enhanced) or absence (non-enhanced) of diluted CHIKV patient plasma containing total IgG at a concentration of 1.8 ± 1.45 μg/ml. (**b**) Level of infection and (**c**) viral RNA load were determined at 24 and 48 hpi. Level of infection was determined by the amount of Zs-Green positive cells and is expressed as fold enhancement relative to the non-enhanced infection controls. Data shown are mean ± SD from 4 independent experiments by Mann-Whitney *U* test (*P = 0.0317 for level of infection at 24 hpi). (**d**) FcγRs expression in RAW264.7 cells was determined by flow cytometry. CHIKV (moi 10) was used to infect RAW264.7 cells (2 × 10^6^ cells per infection) in the presence (enhanced) or absence (non-enhanced) of diluted CHIKV-specific mice sera at concentration of ~2 µg/ml. (**e**) Detection of CHIKV antigen and (**f**) viral RNA load were determined in these infected cells at 0, 6 and 12 hpi. Level of CHIKV antigen detected is expressed as fold enhancement relative to non-enhanced infection controls. Data shown are mean ± SD from 3 independent experiments by parametric unpaired *t* test (*P = 0.0341 and 0.0350 for 0 and 6 hpi respectively for level of infection in RAW264.7 cells; ***P = 0.0006 for 6 hpi and *P = 0.0269 for 12 hpi for viral RNA load in infected RAW264.7 cells).

Macrophages have been hypothesized to act as a possible cellular vehicle and reservoir for virus dissemination and persistence^37–39^. Using human monocytes-derived macrophages (MDMs), which express high levels of FcγRI and II (Fig. 3a), antibody-mediated enhancement of CHIKV infection was investigated with diluted patients’ plasma (total IgG concentrations of 1.8 ± 1.45 µg/ml. In this experiment, infection was once again performed with the Zs-Green tagged CHIKV variant. It was observed that elevated levels of ZS-green positive cells were being detected at 24 hpi (Fig. 3b). Likewise, it was also increased in primary monocytes isolated from the same donor (Fig. S2a). Despite the increased level of Zs-green positive cells, viral RNA load remained unchanged (Fig. 3c and S2b). Similarly, the enhancement observed was mediated via FcγRII, as blocking with FcγRII-specific antibodies completely abrogated infection (Fig. S3).

### Antibody-mediated enhancement of CHIKV infection in mouse macrophages

Before studying the impact of CHIKV antibody-mediated enhancement in mice, *in vitro* CHIKV infections were first performed in the RAW264.7 mouse macrophage cell line. RAW264.7 cells have been used in several studies to investigate the effects of antibody-mediated enhancement of infection in RRV^31,32^, a closely related alphavirus. RAW264.7 cells have high levels of FcγRII/III (Fig. 3d). As a result, an increased in CHIKV infection was observed at 6 hpi in the presence of sub-neutralizing levels of CHIKV-specific mouse sera containing ~2 μg/ml of total IgG antibodies (Table 1) (Fig. 3e). The increased in CHIKV infection was further complemented with higher viral RNA load (Fig. 3f).

**Figure 4:**
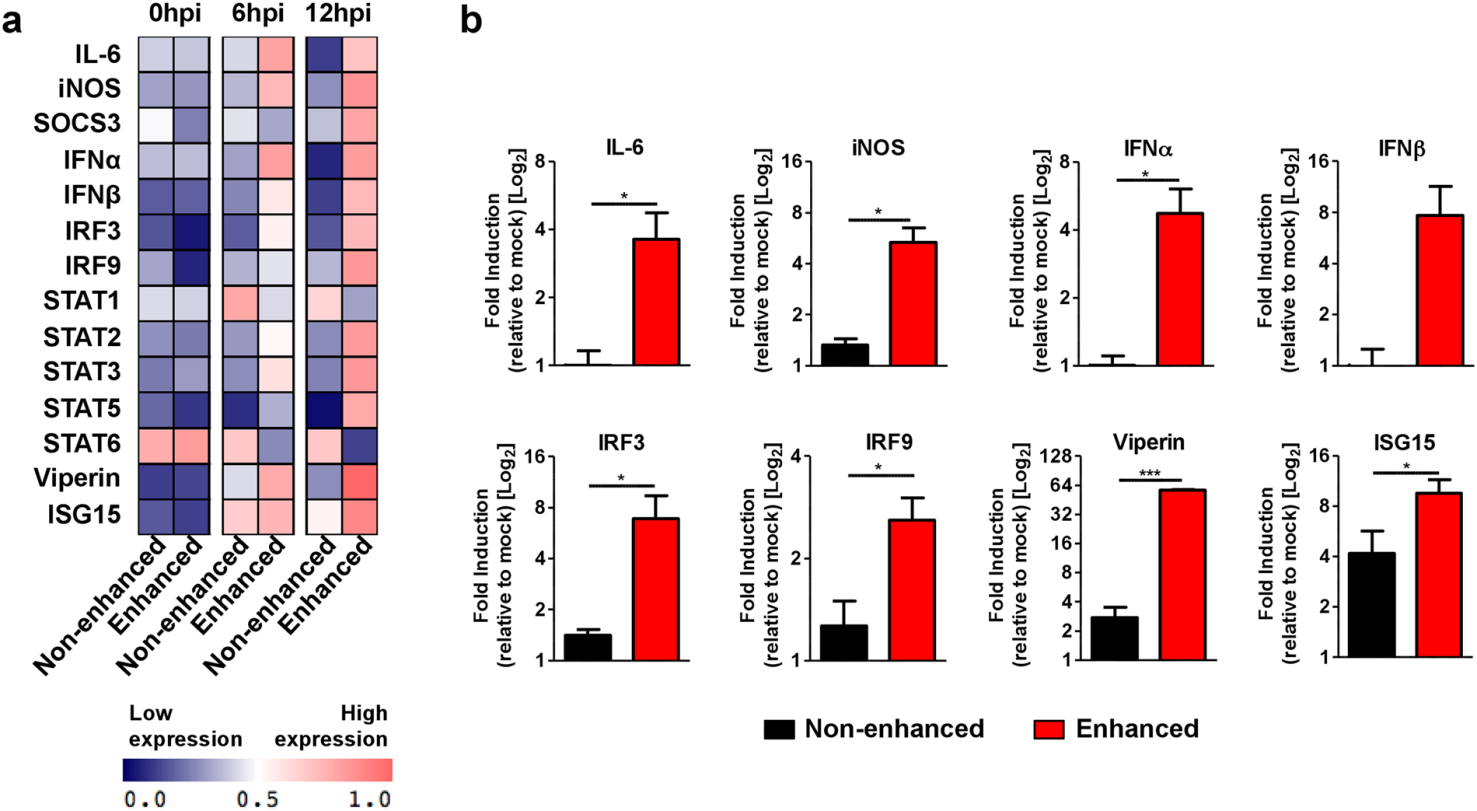
Levels of Type I IFN and pro-inflammatory genes upon CHIKV infection under enhancing conditions. RAW264.7 cells (2 × 10^6^ cells per infection) were infected with CHIKV (moi 10) under both enhancing and non-enhancing conditions and infected cells were harvested at 0, 6 and 12 hpi for gene expression study on selected pro-inflammatory and Type I IFN related genes by qRT-PCR. (**a**) Signature of gene expression across all stipulated time points displayed as a two-way clustered heat plot. Data are normalized to mock-infected controls. (**b**) Elevated expression of key mediators during enhanced CHIKV infection at 12 hpi. Data are normalized relative to mock-infected controls. Data are presented as mean ± SD from 3 independent experiments by parametric unpaired *t* test (*P = 0.0299 for IL-6; *P = 0.0117 for iNOS; *P = 0.0184 for IFNα; *P = 0.0498 for IRF3; *P = 0.0259 for IRF9; *P < 0.0001 for Viperin; *P = 0.0466 for ISG15).

Given that enhanced CHIKV infection in RAW264.7 cells led to both increased levels of CHIKV antigen detected and viral RNA load, the consequences of this enhancement was further studied via gene expression analyses on the Type-I Interferon (IFN) and pro-inflammatory pathways (Fig. 4a). Expectedly, high levels of several pro-inflammatory and anti-viral genes such as IL-6, iNOS, IFNα, IFNβ, IRF3, IRF9, Viperin and ISG15 were detected starting at 6 hpi when CHIKV infection was enhanced (Fig. 4b).

### Antibody-mediated enhancement leads to increased infection and exacerbation of disease severity in CHIKV-infected adult mice

**Figure 5:**
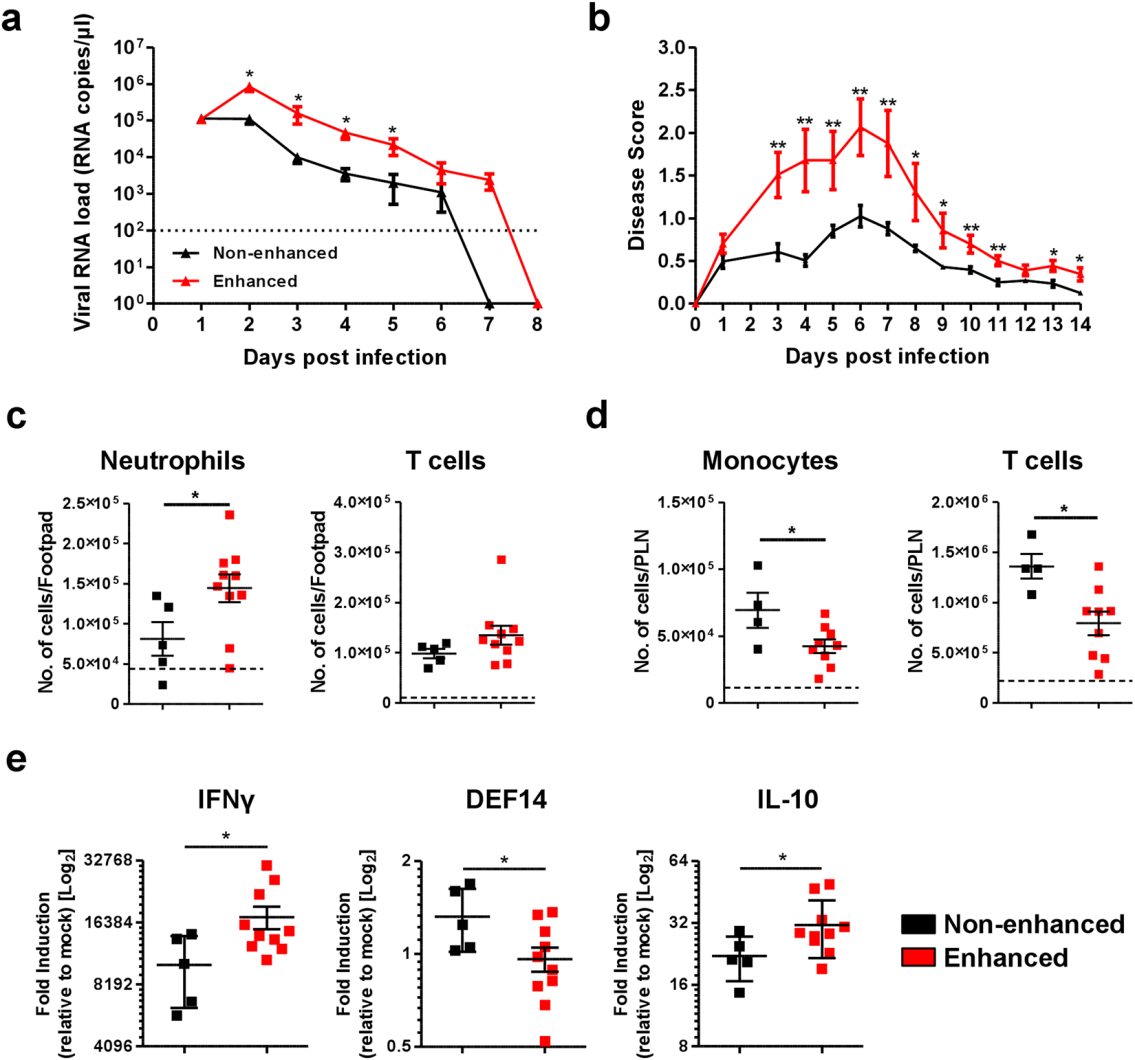
Exacerbation of joint inflammation in adult mice. (**a**,**b**) Three-weeks old, C57BL/6 WT female mice (n = 5 animals per group) were infected via footpad inoculation with 10^6^ PFU of CHIKV. Immediately, these mice were administered intraperitoneally with ~2 µg/ml (total IgG concentration) mice sera from CHIKV-infected mice (enhanced) or PBS (non-enhanced). Daily assessment of (**a**) viremia and (**b**) disease score were performed. All Data are presented as mean ± SD by Mann-Whitney *U* test (*P = 0.0278, *P = 0.0159, *P = 0.0159 and *P = 0.0297 for 2, 3, 4 and 5 dpi viremia respectively; **P = 0.0040, **P = 0.0079, **P = 0.0060, **P = 0.0079, **P = 0.0079, *P = 0.0104, *P = 0.0172, **P = 0.0040, **P = 0.0060, *P = 0.0106 and *P = 0.0106 for 3, 4, 5, 6, 7, 8, 9, 10, 11, 13 and 14 dpi disease score respectively). Immune-phenotyping was performed for (**c**) footpad and (**d**) popliteal lymph node on 6 dpi. Results are displayed as number of neutrophils, CD4+ T cells and monocytes per organ from 4 to 10 animals per group. (**e**) Total RNA was extracted from the joint footpads of infected mice (n = 5 to 10 animals per group) and qRT-PCR was performed to detect for the expression of crucial immune genes. Gene expression data are expressed as fold expression relative to the mock-infected mice. Mice sacrificed for immune-phenotyping and gene expression studies were infected as described for (**a**,**b**). All Data are presented as mean ± SD by Mann-Whitney *U* test (*P = 0.0186, *P = 0.0326 and *P = 0.0185 for neutrophils, monocytes and CD4+ T cells infiltration into respective organs; *P = 0.0276, *P = 0.0376 and *P = 0.0376 for IFNγ, DEF14 and IL-10 expression respectively).

To further establish and evaluate the contribution of antibody-mediated enhancement in CHIKV infection, *in vivo* infections were performed in two different and well-characterized CHIKV mouse models that mimic disease in humans^40–42^. Using the joint footpad model^41–48^, 3 weeks old wild-type (WT) C57BL/6 mice were inoculated with 10^6^ plaque forming unit (PFU) of CHIKV in the right footpad, followed by intra-peritoneal administration of diluted CHIKV-specific mice sera containing total IgG at a concentration of ~2 μg/ml. Disease progression was monitored daily for viremia and degree of joint inflammation at the footpad. Mice infected under enhancing conditions had significantly higher viral RNA load during the acute phase of the disease (Fig. 5a) and endured a more pronounced inflammation at the infected joint footpad (Fig. 5b). Tissue immune-phenotyping (Fig. S4) further revealed higher levels of infiltration of neutrophils and possibly CD4+ T cells into the joint footpad at 6 days post-infection (dpi) during enhanced CHIKV infection (Fig. 5c). Moreover, a much lower number of CD4+ T cells and monocytes were present in the popliteal lymph node (pLN) draining the right footpad (Fig. 5d), indicating the possible egress of these cells from the pLN into the circulation or infected tissues during enhanced infection. Gene expression studies also revealed higher levels of IFNγ as well as IL-10 in the inflamed footpad during enhanced infection. This was coupled with a lower expression of β-defensin 14 (DEF14) (Fig. 5e).

**Figure 6:**
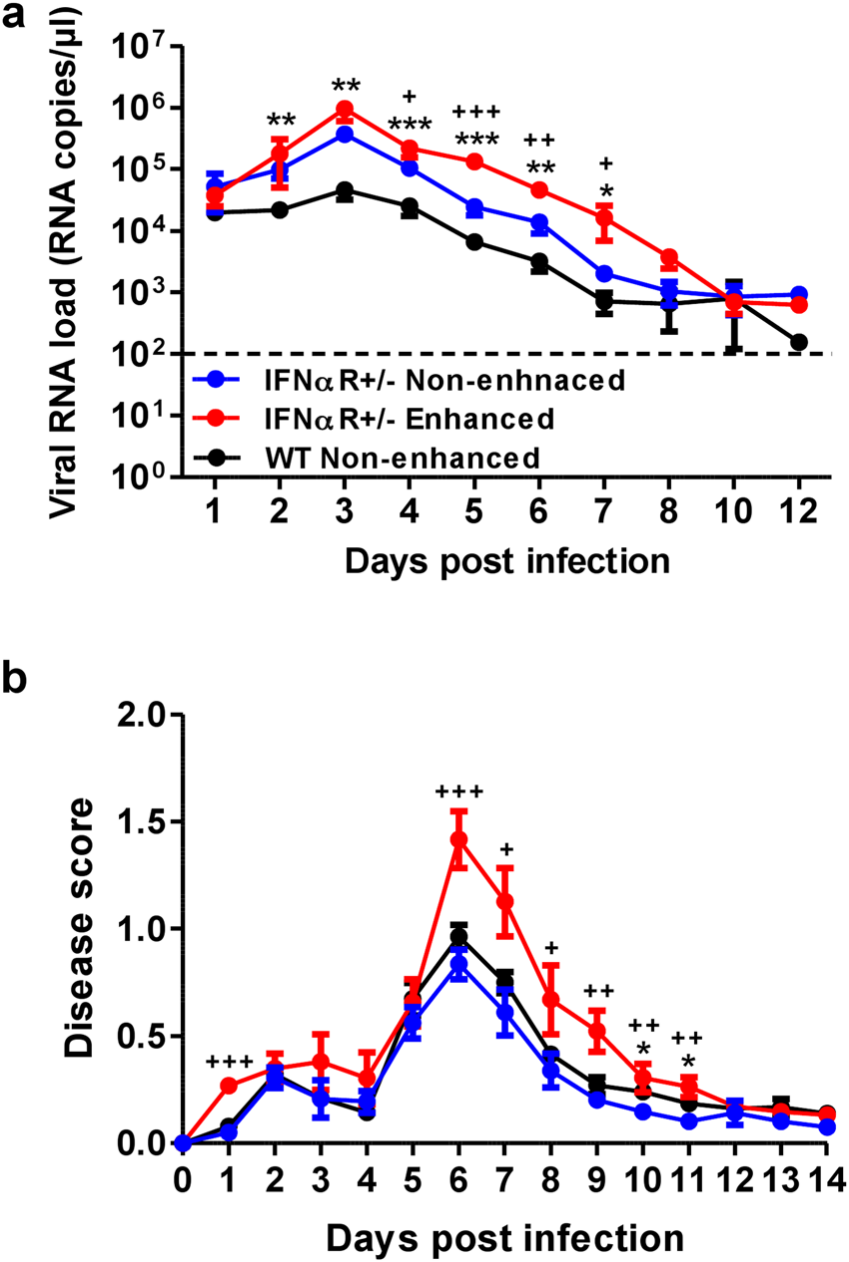
Aggravated disease outcome in CHIKV-infected IFNαR+/− mice. Three-weeks old IFNαR+/− female mice (n = 7 to 11 animals per group) were infected via footpad inoculation with 10^6^ PFU of CHIKV. Immediately, these mice were administered intraperitoneally with ~2 µg/ml (total IgG concentration) mice sera from CHIKV-infected mice (enhanced) or PBS (non-enhanced). Daily assessment of (**a**) viremia and (**b**) disease score were performed. Data are presented as mean ± SD by Mann-Whitney *U* test (**P = 0.0027, **P = 0.0011, ***P = 0.0009, ***P = 0.0005, **P = 0.0017 and *P = 0.0159 for 2, 3, 4, 5, 6 and 7 dpi viremia respectively; ^+^*P* = 0.0125, ^+++^*P* < 0.0001, ^++^*P* = 0.0015 and ^+^*P* = 0.0277 for 4, 5, 6 and 7 dpi viremia respectively; *P = 0.0109 and *P = 0.0306 for 10 and 11 dpi disease score respectively; ^+++^*P* < 0.0001, ^+++^*P* = 0.0006, ^+^*P* = 0.0109, ^+^*P* = 0.0277, ^+^*P* = 0.0031, ^+^*P* = 0.0095 and ^+^*P* = 0.0031 for 1, 6, 7, 8, 9, 10 and 11 dpi disease score respectively. (*) indicates significance comparing between CHIKV-infected IFNαR+/− (blue) and WT animals (black); (+) indicates significance comparing between IFNαR+/− mice infected under enhanced (red) and non-enhanced (blue) conditions.

In a second model, intradermal CHIKV infection in IFNαR+/− mice leads to the development of a mild infection, with CHIKV targeting the muscles, joint and skin fibroblasts^40^. Utilizing this model, 3 weeks old IFNαR+/− mice were infected with 10^6^ PFU CHIKV via the joint footpad to assess the outcome of antibody-mediated enhancement. While a similar degree of joint footpad inflammation (Fig. 6a) was observed in IFNαR+/− mice as compared to WT mice, higher viremia was detected in the IFNαR+/− animals (Fig. 6b). However, when CHIKV infection was performed in the presence of diluted CHIKV-specific mice sera at a concentration of ~2 μg/ml total IgG, increased viremia (Fig. 6a) and more pronounced joint footpad inflammation (Fig. 6b) was observed in the IFNαR+/− mice. These observations reinforce the significance of sub-neutralizing concentrations of CHIKV-specific antibodies in enhancing CHIKV infection and aggravating disease pathology.

### CHIKV-specific polyvalent human antibodies aggravate CHIKV infection in young mice, leading to death

**Figure 7:**
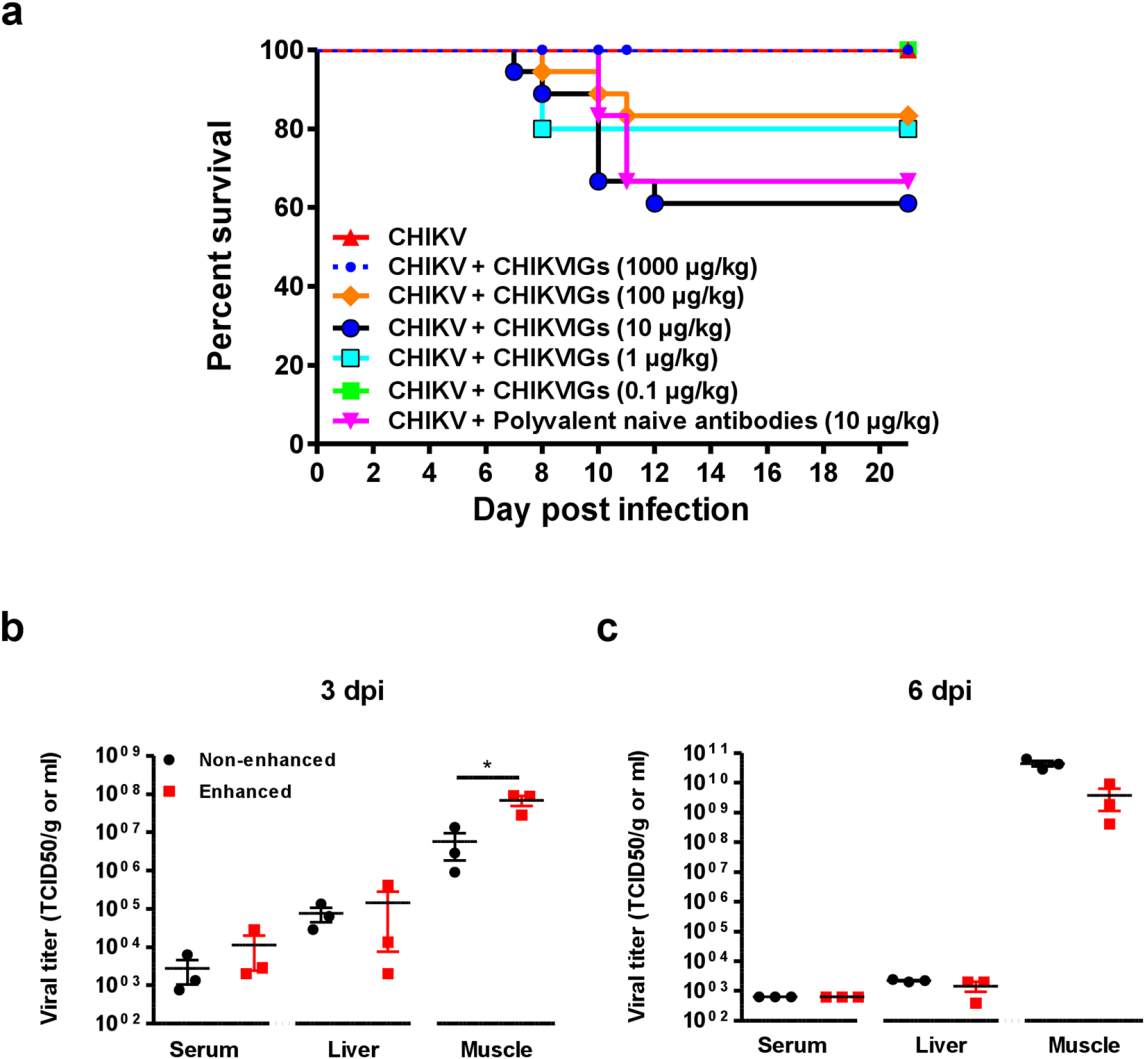
Enhanced infection-associated lethality in young mice. (**a**) 11-day old C57BL/6 (female, n = 10 animals per group) were inoculated intradermally with 10^6^ PFU of CHIKV-21. Immediately after, these mice were given intraperitoneally either human naive polyvalent antibodies or purified CHIKVIGs at different concentrations. Survival of these animals was monitored over a period of 3 weeks. (**b**,**c**) 11 days old C57BL/6 (female, n = 3 animals per group) were infected as described in (**a**) but were intraperitoneally given purified human CHIKVIGs at 10 µg/kg. Control animals were given 10 µg/kg of human naive polyvalent antibodies. Viral RNA load in the serum, liver and muscle were determined at both (**b**) 3 and (**c**) 6 dpi. All data are presented as mean ± SD. *p < 0.05 by parametric unpaired *t* test.

It has been demonstrated that prophylactic administration of human purified polyvalent immunoglobulins G (IgG) containing anti-CHIKV antibodies (CHIKVIGs) protected susceptible IFNα/βR−/− adult animals and wild-type neonates during CHIKV infection^49^. To assess if these antibodies could exacerbate CHIKV disease severity at sub-neutralizing concentrations, different concentrations of CHIKVIGs were administered intra-peritoneally to CHIKV-infected 11 days old C57BL/6 WT mice at the ventral thorax^40^ (Fig. 7). Mortality was observed in mice receiving CHIKVIGs at concentrations of 1–100 µg/kg (Fig. 7a), whereas mice receiving either 1000 µg/kg or 0.1 µg/kg of CHIKVIGs survived the infection. Unexpectedly, approximately 30% of mice receiving a control non-CHIKV-specific polyvalent IgG also died from infection (Fig. 7a). Nevertheless, viral RNA load in serum, liver and muscles was determined in CHIKV-infected mice receiving 10 µg/kg of CHIKVIGs. It was observed that enhanced CHIKV infection led to a more significant viral burden in the muscles at 3 dpi (Fig. 7b), but not at 6 dpi (Fig. 7c). Taken together, these findings highlight that enhanced CHIKV infection is detrimental in young animals.

## Discussion

**Figure 8:**
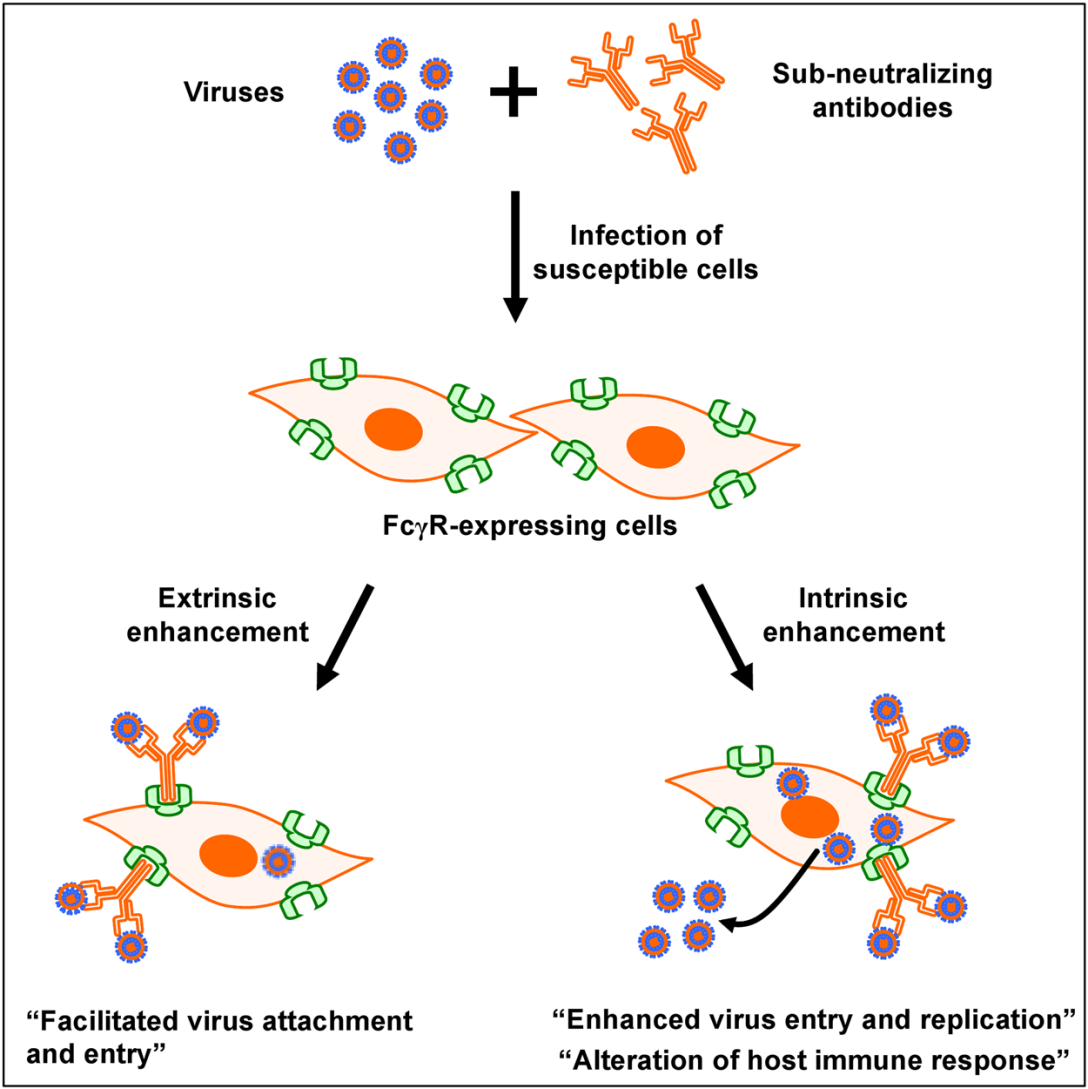
Impact of antibody-mediated enhancement in CHIKV infection. Virus-antibody complexes are formed when antibodies are present in sub-neutralizing concentrations and are taken up by cells expressing FcγRs via either extrinsic or intrinsic pathways. Extrinsic pathways result in increased virus attachment and possibly entry with no visible benefits to virus replication. Whereas in intrinsic pathways, it can lead to enhanced anti-inflammatory or enhanced pro-inflammatory infection. Enhanced anti-inflammatory infection downplays the inflammatory response associated with normal infection and instead upregulates the anti-inflammatory response leading to heightened infection and viral replication. In enhanced pro-inflammatory infection, it leads to an increased in active infection, without negative suppression of pro-inflammatory immune response.

This study provides the first documentation of antibody-mediated enhancement in CHIKV infection *in vitro* and in relevant *in vivo* mouse models. Despite the increased detection of CHIKV antigen in primary human monocytes, B cells and MDMs when infection was performed in the presence of sub-neutralizing levels of CHIKV-specific antibodies, viral replication was not increased. Antibody-mediated enhancement in viral infections has been reported to mostly involve FcγRs^22^. The involvement of FcγRs in enhancing CHIKV infection may lead to two outcomes: the extrinsic and intrinsic pathways (Fig. 8). In the extrinsic pathway, virus attachment and possibly entry is facilitated without much significance to viral replication and manipulation of host immune responses^22,50^. Results obtained with infection in the whole blood, primary human monocytes, B cells and MDMs supported that the extrinsic pathway is at play. The increase detection of Zs-Green signal in MDMs during the later time points is suggestive of viral entry and initiation of viral replication, as the Zs-Green is cloned under a duplicated sub-genomic promoter^42,51^. However, no further increase in both Zs-Green signal and viral RNA load may suggest that enhanced CHIKV infection do not necessary augments viral replication. This quick shutdown of viral replication may actually indicate a putative mechanism employed by CHIKV for chronic persistence. While it is known that CHIKV could persist chronically using macrophages as possible cellular reservoirs^38,39^, its exact mechanism is not known. As such, the restricted replication observed in monocytes and MDMs could be a form of immune subversion by the virus to achieve chronic persistency^52^.

On the other hand, the intrinsic pathway typically results in increased virus entry and replication, often accompanied by the alterations of host immune responses^31,32,53–56^ (Fig. 8). Intriguingly, enhanced CHIKV infection in murine RAW264.7 macrophages was even more prominent when the virus started replicating (6 hpi). Subsequently, this led to the high expression of several pro-inflammatory and anti-viral genes. This observation is enthralling as similar studies in DENV and RRV reported the down-regulation of these pro-inflammatory responses^31,32,53–56^. While the data in this report differs, it opens up another compelling perspective in which enhanced CHIKV infection in susceptible cells can lead to augmented virus entry and replication, resulting in an active pro-inflammatory response from the infected cells (Fig. 8). While the consequences of the elevated immune response remains to be investigate, it is plausible that CHIKV manipulates this immune outcome to its own advantage to achieve chronicity, as observed for other viral infections^57^.

Whether antibodies are enhancing or neutralizing depends on numerous factors such as possible future infection with closely-related viruses (e.g., other alphaviruses), the virus strain, virus titer and concentrations, epitopes specificities, isotypes and FcγRs-binding affinities of the antibodies^22,58–60^. Patient plasma and mice serum samples used in this study at a low dilution factor have been previously shown to be strongly neutralizing^44,46,61,62^ and recognized epitopes located mainly on the CHIKV E2 glycoprotein^44,46^. Furthermore, these CHIKV-specific antibodies were of IgG3 isotype^62^, and hence capable of moving across the placenta^63^. It remains to be seen if infants born to mothers infected with CHIKV during their pregnancy may suffer from a more severe disease due to the low levels of maternal-acquired CHIKV-specific antibodies. It was reported that newborn mice infected with DENV in the presence of maternally acquired anti-DENV antibodies had a more severe disease outcome^64^.

Using the joint footpad mouse model of CHIKV infection^41–48^, disease severity was greatly enhanced in both WT and IFNαR+/− mice upon passive administration of low levels of CHIKV-specific antibodies. The exact mechanisms of this phenomenon remains to be fully elucidated. However as presented in this study, the disruption of host immune response could be a crucial driving force behind the augmented disease severity. This brings caution to the phenomenon that sub-neutralizing concentrations of virus-specific antibodies can enhance severity of infection and advocates the need for careful vaccine design and extensive pre-clinical trials. In fact, it was reported that CHIKV severity was increased in vaccinated mice that presented sub-optimal immune responses^65^. Unfortunately, it was also observed that a proportion of mice receiving the naïve human polyvalent antibodies succumbed to CHIKV infection. This was definitely an unexpected observation and could be due to the presence of unknown artifacts present in the control antibodies that resulted in this outcome. These naïve polyvalent antibodies had previously been shown not to exhibit any immuno-reactivity against CHIKV^49^.

To conclude, the relevance of antibody-mediated enhancement in aggravating viral disease severity should not be underestimated. The recent 2016 outbreaks of ZIKV infections across the globe and the potential of anti-DENV antibodies cross-reacting with ZIKV^33^ highlights the relevance of this anomaly. Therefore, there is a need to expand research efforts in the understanding and prevention of antibody-mediated enhancement in viral infections.

## Methods

### Ethics approval

This study was reviewed and approved by the institutional review board at the National Healthcare Group with Domain Specific Review Board no. DSRE E/08/414. Written informed consent was obtained from all CHIKF patients prior to the collection of samples. Human blood samples were obtained from healthy donors with written informed consent in accordance with the guidelines from the Health Sciences Authority of Singapore (study approval number: NUS IRB number 10–250). Animal studies protocols were approved: (1) by the Institutional Animal Care and Use Committee (IACUC) of the Agency for Science, Technology and Research (A*STAR) (IACUC number: 151018); and (2) by the Institut Pasteur Animal Ethics Committee according to project #2014-0019 using level 3 isolators.

### Patient plasma samples

Plasma samples from patients, who were admitted with acute CHIKF to the Communicable Disease Centre at Tan Tock Seng Hospital (CDC/TTSH), during the outbreak from 1 August to 23 September 2008^8,9,35^ were used in this study. These plasma have previously been characterized^44,61,62,66^. In this study, plasma samples collected 2–3 months post-illness onsets were used. All patient plasma were aliquoted and stored at −80 °C. Plasma samples were also collected from 8 healthy volunteers as controls.

### Primary cells (B cells and monocytes) isolation

Peripheral blood mononuclear cells (PBMCs) were extracted from 50 ml of donors’ blood as described previously^37^. Extracted PBMCs were subjected to either B cells or monocytes isolation using B cell Isolation Kit II and Monocytes Isolation Kit II (Miltenyi Biotec) respectively following manufacturer’s instructions. Purity was > 95% as verified by flow cytometry.

### Cell culture

African green monkey kidney epithelial cells (Vero-E6) and mouse macrophage cell line RAW264.7 cells were maintained in Dulbecco’s Modified Eagle Medium (DMEM) (Gibco) supplemented with 10% Fetal Bovine Serum (FBS) (Gibco). Human B lymphocytic ST486 and Fcγ Receptors (FcγRs) over-expressing ST486 cell lines were cultured in Roswell Park Memorial Institute medium (RPMI 1640) supplemented with 10% FBS. *Aedes* albopictus monolayer (C6/36) cells were cultured in Leibovitz’s medium (L-15) (Gibco) supplemented with 10% FBS. Extracted primary human cells were maintained in Iscove’s Modified Dulbecco’s Medium (IMDM) (Hyclone) supplemented with 10% Human serum (HS) (I-DNA Biotechnology). All cultures were incubated at 37 °C with 5% CO_2_ supplied with the exception of C6/36 which was incubated at 28 °C with no CO_2_ supplied. All media and reagents were tested negative for endotoxins.

### MDMs differentiation

Isolated monocytes were differentiated into MDMs as described^67,68^ with some modifications. Briefly, isolated monocytes were allowed to differentiate over a period of 5 days in IMDM (Hyclone) supplemented with 10% HS (I-DNA Biotechnology). A change of fresh medium was performed on the third day, in which the non-adhering cells (presumably non-differentiated monocytes or dead cells) would have been removed, leaving behind the adhering MDMs.

### Virus stocks

CHIKV-SGP11 was isolated from an outbreak in Singapore in 2008 and prepared in Vero-E6 cells as described^37^. This was used for all *in vitro* experiments involving isolated primary human monocytes, B cells and the RAW264.7 cells. For the whole blood and MDMs (together with their corresponding precursor monocytes), a full-length CHIKV infectious clone based on the CHIKV LR2006-OPY1^69^ expressing the Zs-Green protein was used. CHIKV-SGP11 was further propagated in C6/36 cells and purified by ultra-centrifugation^44^ before being used in infection studies in 3 weeks old mice. In infection studies in 11 days old mice, CHIKV-21 isolate obtained from a patient during the 2005–2006 outbreak of CHIKV infection in La Réunion was used^49^.

### *In vitro* FcγR expression assay

Cells were first stained with 10 μl of Human FcγR blocking reagent (Miltenyi Biotec), before staining with the following anti-FcγR antibodies and respective fluorophore isotype controls following manufacturer’s recommendations: FITC-conjugated mouse anti-human CD64 (eBioscience), APC-conjugated mouse anti-human CD32 (eBioscience), PE-conjugated mouse anti-human CD16 (eBioscience), mouse IgG1κ isotype control FITC (eBioscience), mouse IgG1κ isotype control APC (eBioscience) and mouse IgG1κ isotype control PE (eBioscience). For RAW264.7 cells, staining was performed with FITC-conjugated rat anti-mouse FcγRI and anti-mouse FcγRII antibodies (R&D). Data were acquired with BD FACS Calibur (BD Biosciences) using BD CellQuest Pro software (BD Biosciences). Results were analyzed with FlowJo (version 10) software (Tree Star).

### *In vitro* CHIKV infection assay

CHIKV (moi 10) was first incubated with diluted heat-inactivated (56 °C for 30 min) CHIKV-specific patient plasma or animal sera in serum-free IMDM (Hyclone) or DMEM (Gibco) respectively, for 2 h on a shaking heat-block (37 °C; 350 rpm) before being used to infect the respective primary cells or cells lines (2 × 10^6^ cells per infection) for 1.5 h in a 37 °C incubator, with atmosphere of 5% (v/v) CO_2_. Virus overlay was removed and cells were washed once with appropriate serum-free medium before they were re-suspended in appropriate complete medium. Cells were further incubated at 37 °C, with atmosphere of 5% (v/v) CO_2,_, before being harvested at indicated time points. During harvesting, 140 μl of infected cell suspension was aliquoted for viral RNA extraction. For gene expression studies, an aliquot of cell suspension was spun down and the resultant cell pellet was dried before being stored in −80 °C for downstream total RNA extraction. Remaining cells were collected by centrifugation and fixed with FACS lysing buffer (BD Biosciences) and stored for downstream staining procedures. Mock and non-enhanced control infections were performed by incubating cells with serum-free DMEM (Gibco) and viruses respectively. For *ex vivo* infection in human whole blood, Zs-Green tagged CHIKV variant (2 × 10^7^ PFU) was first incubated with diluted heat-inactivated CHIKV-specific patient plasma in serum free IMDM (Hyclone) for 2 h on a shaking heat-block (37 °C; 350 rpm) before being added to 1 ml of fresh citrate whole blood. 1 ml of whole blood typically contains between 1.5–2 × 10^6^ leukocytes. Infection was incubated in 37 °C, with atmosphere of 5% (v/v) CO_2_ until being harvested at the indicated time points. During harvesting, the mixture was spun down and 140 μl of plasma was obtained for viral RNA extraction. Red blood cells were subseqeuntly lyzed and cell pellet washed and subsequently fixed with FACs lysing buffer (BD Biosciences and stored for downstream staining procedures. Mock and non-enhanced control infections were performed by incubating cells with serum-free IMDM (Hyclone) and viruses respectively. Results are expressed as fold enhancement relative to non-enhanced infections. Fold enhancement is calculated according to the equation: Fold enhancement = (Level of CHIKV antigen detected from enhanced infection group / level of CHIKV antigen detected from non-enhanced infection group).

### *In vitro* FcγR blocking assay

FcγR blocking agent (Miltenyi Biotec) was added to the cells for 30 min prior to CHIKV infection under enhancing conditions as described above. Immediately after the virus overlay was removed, cells were washed and harvested for downstream procedures as mentioned above. For FcγRII blocking, mouse anti-human CD32 antibodies (Stemcell) were used instead. Results are expressed as % infectivity relative to non-treated-enhanced infections. Reduction in infectivity was calculated according to the equation: % infectivity = 100 × Level of CHIKV antigen detected from treated-enhanced infection group / Level of CHIKV antigen detected from non-treated-enhanced infection group).

### Viral RNA load assay

Viral RNA was extracted using QIAamp Viral RNA Mini Kit (QIAGEN), following manufacturer’s instructions. Viral RNA load was subsequently measured using real time quantitative reverse transcription PCR (qRT-PCR) utilizing QuantiTect® Probe RT-PCR kit (QIAGEN) modified from a previously described method to detect negative-strand nsP1 RNA^70,71^.

### Flow cytometry

Fixed cells were permeabilized with FACS permeabilizing solution 2 (BD Biosciences). For detection of CHIKV antigens in primary human cells (B cells, monocytes), staining was done as described previously using a commercially available anti-alphavirus mAb (Santa Cruz; SC-58088)^37,47^. For whole blood and human MDMs, CHIKV infection was directly quantified by the detection of Zs-Green signal under the FITC channel. For detection of CHIKV antigen in RAW264.7 cells, staining was performed with rabbit anti-CHIKV nsP2 antibodies followed by secondary staining with Alexa Fluor 488-conjugated Goat anti-rabbit IgG (H + L) (Invitrogen). For primary human cells, an additional staining step was performed. Surface markers Pacific-Blue-conjugated mouse anti-human CD45, Qdot-605-conjugated mouse anti-human CD19, PerCP Cy5.5 mouse anti-human CD14 and PE-Cy7-conjugated mouse anti-human BDCA2 (all antibodies were from eBiosciences) were stained following manufacturer’s protocol. Human monocytes, B cells and pDCs are defined by the specific surface expression of CD14, CD19 and BDCA2 respectively. Data was acquired using either BD FACS Calibur or BD FACS Canto II (BD Biosciences). Softwares used include BD CellQuest Pro software (for FACSCalibur) and BD FACSDiva software (for FACSCanto II) (BD Biosciences). Gating strategy is shown in Fig. S5. A total of 30,000–50,000 cells were acquired and results were analyzed with FlowJo (version 10) (Tree Star).

### Gene expression

Total RNA from cells was extracted using RNeasy® Mini Kit (QIAGEN) according to manufacturer’s instructions, without any DNAse treatment. Eluted RNA was quantified and diluted to 10 ng/µl before being stored in −80 °C. Gene expression profiling was performed via RT-PCR using QuantiFAST^TM^ SYBR® Green RT-PCR Kit (QIAGEN) as described^47^. Results are expressed as relative fold change in expression level compared to mock infections, as previously described^47^. Gene expression data from infected RAW264.7 cells are displayed by two-way hierarchical clustering generated with Multi Experiment Viewer (version 4.9) (Microarray Software Suite TM4)^72^. Sequences of primers used are displayed in Table S1.

### Animal studies

CHIKV infection in 3 weeks old WT and IFNαR+/− C57BL/6 mice was performed. Briefly, 10^6^ PFU of CHIKV isolate (SGP11), diluted in 30 µl of PBS, were inoculated subcutaneously in the ventral side of the right hind footpad, towards the ankle^42,43,45–48^. Following this, PBS diluted (1:1000) CHIKV-specific mice sera were passively administered intraperitoneally into the infected animals. Control mice were inoculated with PBS alone. Viremia and the degree of footpad inflammation were monitored as described previously^42,43,45–48^. Three weeks old animals were bred and kept under specific pathogen-free conditions in the Biological Resource Centre, A*STAR, Biopolis, Singapore. For *in vivo* model with human polyvalent immunoglobulins, 11 days old female C57BL/6 mice were inoculated intra-dermally in the ventral thorax with 10^6^ PFU CHIKV isolates (CHIKV-21) diluted in 30 µl of sterile PBS. Human polyvalent immunoglobulins (0.1 µg/kg to 1000 µg/kg) were given intraperitoneally following virus inoculation. Control animals were given 10 µg/kg of human naive polyvalent antibodies. Survival of all experimental animals was monitored daily and were handled in accordance with the guidelines described above.

### Extraction of animal tissues

Extractions the joint footpad were performed as previously described^42,43,45,48^. Excised joint footpads were shredded and digested in digestion medium containing dispase (2 U/ml; Invitrogen), collagenase IV (20 mg/ml; Sigma-Aldrich), and DNase I mix (50 mg/ml; Roche Applied Science) in complete RPMI medium for 3 h at 37 °C. Cell debris and skin tissues were removed by passing through 40 μm cell strainer (BD Falcon) followed by RBC lysis (R&D system). Cells were further purified by spinning down in 35% v/v Percoll (Sigma) solution in RPMI prior to staining. The popliteal lymph node (pLN), located at the area to the back of the mice knee joint, was delicately retrieved and briefly digested in 1 ml of digestion medium containing dispase (2 U/ml), collagenase IV (20 mg/ml), and DNase I mix (50 mg/ml) in complete RPMI medium for 30 min at 37 °C. Disintegration of pLN was encouraged by gentle pipetting before passing contents through a 70 μm nylon mesh cloth (Sefar). RBCs were further lyzed with RBC lysis buffer (R&D system) prior to further staining procedures.

For total RNA extraction from excised joint footpad, specimens were first shredded in TRIzol (Invitrogen) with Micro Smash^TM^ (Digital Biology), a micro-homogenizing system, following these conditions: 2 rounds of homogenization at 5000 rpm for 45 s followed by 30 s on ice. Subsequently, the sample was spun to remove the unwanted cell debris. Chloroform was then added to the TRIzol fraction causing a phase separation into the aqueous and organic phase. The aqueous phase, which contains the RNA, was carefully removed into a clean eppendorf tube. It was then further processed with the RNeasy® Mini Kit (QIAGEN) according to manufacturer’s instructions, before being used for gene expression studies as described above. No DNAse treatment was performed during the total RNA extraction.

### Leukocytes profiling

Footpad and pLN cells were resuspended in blocking buffer (1% v/v rat and mouse serum) for 20 min. Live cells were stained with a Live/Dead Fixable Aqua Dead Cell Stain Kit (Invitrogen) for 30 min before staining with the following cell-specific markers following manufacturer’s protocol: APC-Cy7-conjugated rat anti- mouse CD45 (BD Biosciences), PE-Cy7–conjugated rat anti-mouse CD3 (Biolegend), Pacific Blue-conjugated rat anti-mouse CD4 (Biolegend), PE-conjugated rat anti-mouse CD11b (BD Biosciences) and APC-conjugated rat anti-mouse Ly6G (Biolegend) for 20 min at room temperature. Cells were subsequently washed and fixed with 100 µl neat IC fixation buffer (eBioscience) for 5 min. Cells were then washed and resuspended for flow cytometry data acquisition. Data were acquired BD FACSCanto II (BD Biosciences) with FACSDiva software. Analyses were performed using FlowJo (Version 10) (Tree Star).

### Quantification of Total IgG antibodies

The total amount of IgG present in both heat-inactivated CHIKV-infected patient’s plasma and CHIKV-infected mice sera were quantified with IgG Human ELISA Kit and IgG Mouse ELISA Kit (Abcam) respectively following manufacturer’s protocol.

### Statistical analysis

Comparisons between different groups were performed using either non-parametric Mann-Whitney rank sum test or parametric unpaired t-test (two tail analyses), when data follow a normal distribution. Analyzes were performed with GraphPad PRISM (version 6) (GraphPad Software). *P* values of < 0.05 are considered to be statistically significant.

## Electronic supplementary material

Supplementary Materials
