# Supplementary material for: Antibody-mediated enhancement aggravates chikungunya virus infection and disease severity
Source: Sci Rep. 2018 Jan 30;8:1860. doi: 10.1038/s41598-018-20305-4 (PMC5789897; doi:10.1038/s41598-018-20305-4)
Supplement: Supplementary file 1 — Supplementary Materials [file 41598_2018_20305_MOESM1_ESM.pdf]

Scientific Reports

Supplemental Materials

Antibody-mediated enhancement aggravates chikungunya virus infection and disease severity

Fok-Moon Lum[1,2]*, Thérèse Couderc[3,4], Bing-Shao Chia[1,#a], Ruo-Yan Ong[1,#b], Zhisheng Her[1,#c], Angela Chow[5], Yee-Sin Leo[5], Yiu-Wing Kam[1], Laurent Rénia[1], Marc Lecuit[3,4,6] and Lisa F.P. Ng[1,2,7]*

[1]Singapore Immunology Network, Agency for Science, Technology and Research (A*STAR), Singapore 138648, Singapore

[2]Department of Biochemistry, Yong Loo Lin School of Medicine, National University of Singapore (NUS), Singapore 117596, Singapore

[3]Biology of Infection Unit, Institut Pasteur, Paris 75015, France

[4]Inserm U1117, Paris 75015, France

[5]Institute of Infectious Disease and Epidemiology, Tan Tock Seng Hospital, Singapore 308433, Singapore

[6]Paris Descartes University, Sorbonne Paris Cité, Institut Imagine, Necker-Enfants Malades University Hospital, Division of Infectious Diseases and Tropical Medicine, APHP, Paris 75743 France

[7]Institute of Infection and Global Health, University of Liverpool, Liverpool L69 7BE, United Kingdom

[#a]Department of Microbiology and Immunobiology, Harvard Medical School, Boston, Massachusetts 02115, United States

[#b]School of Life Sciences, University of Dundee, Dundee DD1 5EH , United Kingdom

[#c]Institute of Molecular and Cell Biology, Agency for Science, Technology and Research (A*STAR), Singapore

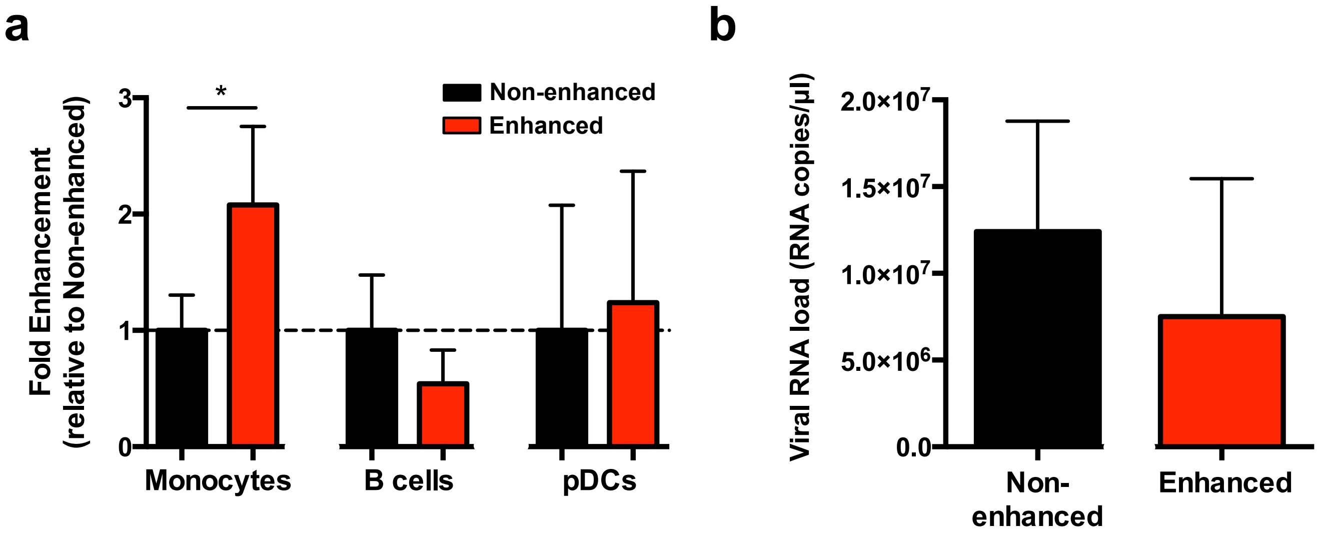

Figure S1: Enhanced CHIKV infection in human whole blood. Human whole blood was infected with Zs-Green tagged CHIKV (2×10^7 PFU) either in the presence (enhanced) or absence (non-enhanced) of diluted CHIKV patient plasma containing total IgG at a concentration of 1.8 ± 1.45µg/ml. (**a**) Level of infection and (**b**) viral RNA load were determined 24 hours post-infection (hpi). Level of CHIKV infection was determined by the amount of Zs-Green positive cells and is expressed as fold enhancement relative to the non-enhanced infection controls. Data shown are mean ± SD from 5 independent donors by Mann-Whitney *U* test (*P = 0.0115).

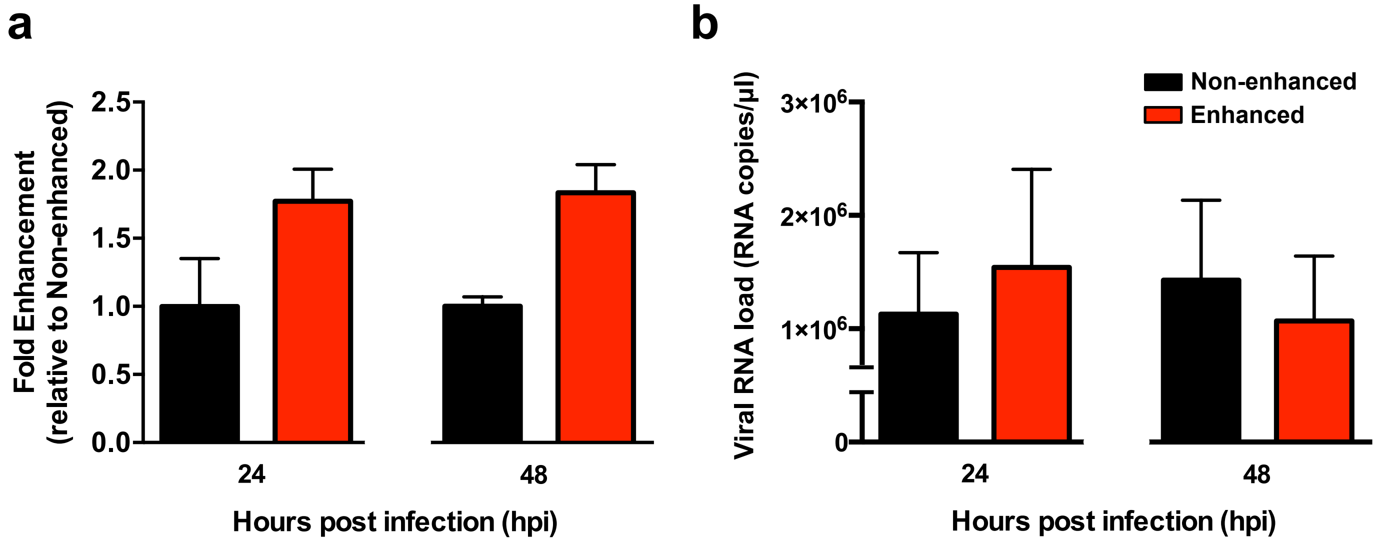

Figure S2: CHIKV infection enhancement in primary human monocytes. Isolated primary human monocytes (2×10^6 cells) were infected with Zs-Green tagged CHIKV (moi 10) either in the presence (enhanced) or absence (non-enhanced) of diluted CHIKV patient plasma containing total IgG at a concentration of 3.6 ± 2.9µg/ml. (**a**) Level of infection and (**b**) viral RNA load were determined at 24 and 48 hpi. Level of CHIKV infection was determined by the amount of Zs-Green positive cells and is expressed as fold enhancement relative to the non-enhanced infection controls. Data shown are mean ± SD from 4 independent experiments.

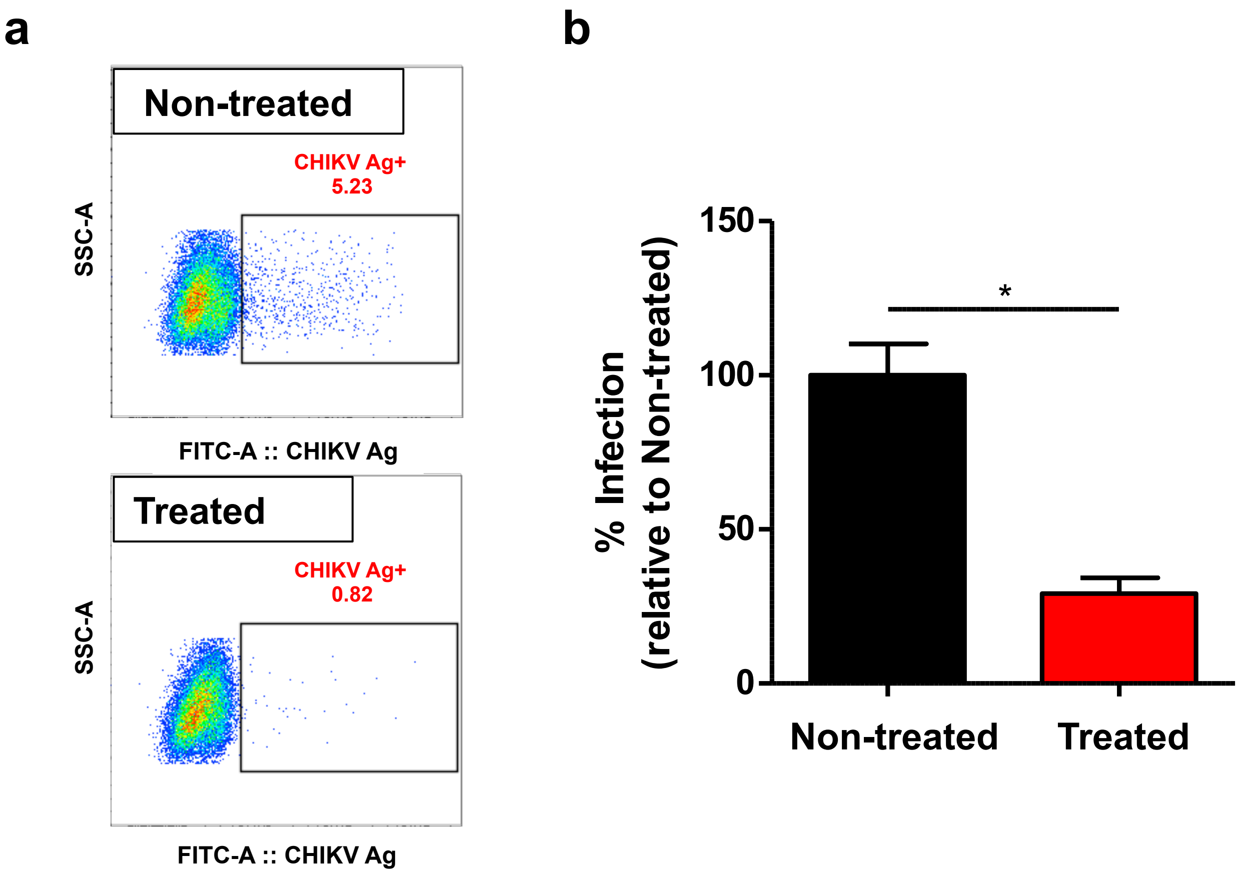

Figure S3: Enhanced CHIKV infection in human monocytes-derived macrophages (MDMs) is mediated by FcγRII. MDMs (2×10^6 cells) were treated with 10µg of FcγRII blocking antibody prior to infection with Zs-Green tagged CHIKV (moi 10) in the presence of CHIKV patient plasma containing total IgG at a concentration of 1.8 ± 1.45µg/ml. (**a**) FACs plot showing the decrease in the amount of CHIKV antigen detected from one representative experiment at 24 hours post-infection (hpi). (**b**) Percentage of infection is expressed as level of CHIKV antigen detected relative to the non-treated-enhanced infection controls. Data are presented as mean ± SD from 4 independent experiments by Mann-Whitney *U* test (*$P = 0.0143$).

Fig S3_Lum *et al.*, 2017

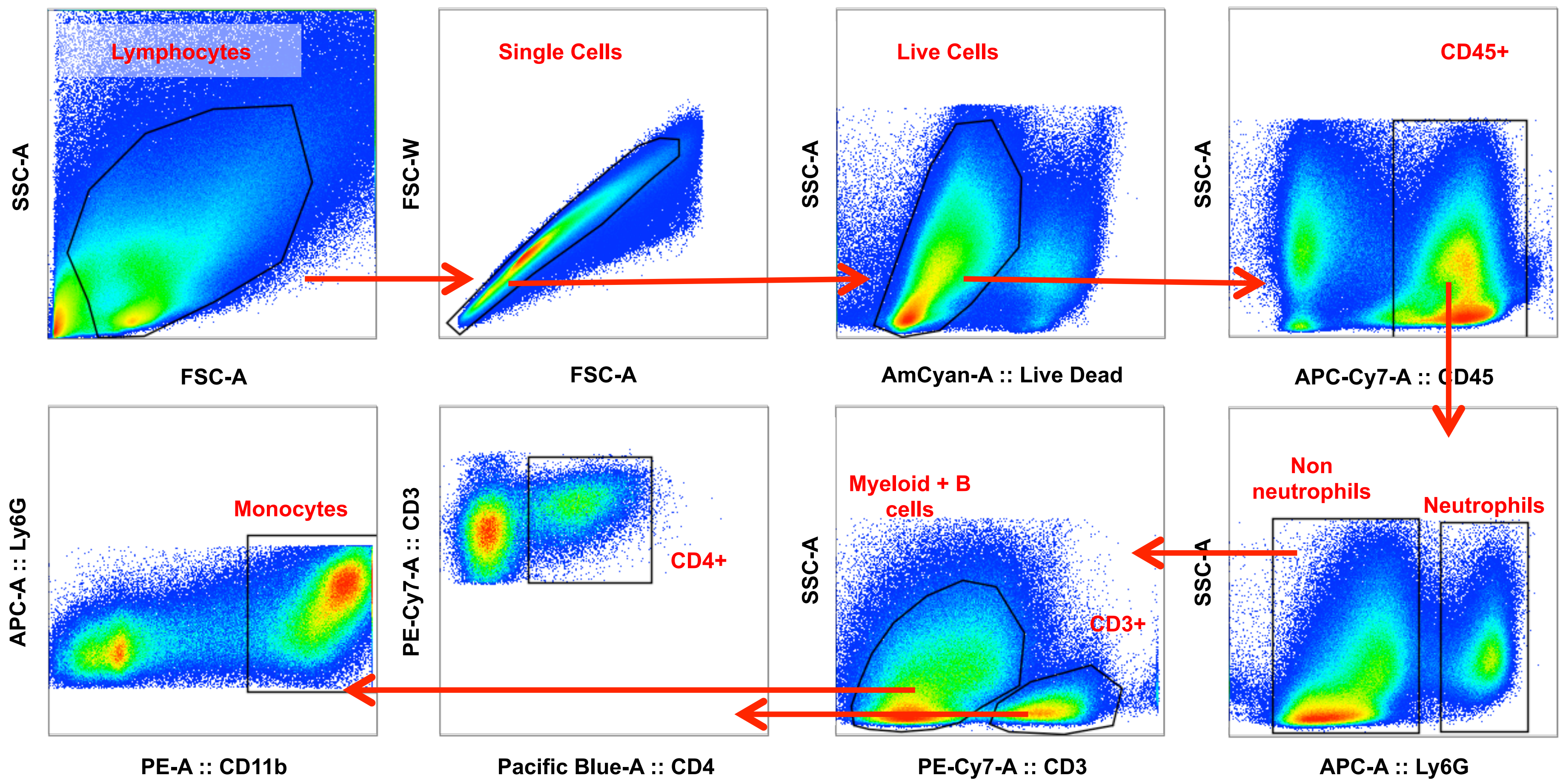

Figure S4: Immunephenotyping of CHIKV infected WT mice. Three-weeks old, C57BL/6 WT female mice were infected via footpad inoculation with 10^6 PFU of CHIKV. Immediately, these mice were administered intraperitoneally with ~2µg/ml sera from CHIKV-infected mice (enhanced) or PBS (non-enhanced). Gating stratefy of immune-phenotyping illustrated using samples extracted from the CHIKV-infected footpad is shown here. Strategy described is used for all analyzes.

Fig S4_Lum *et al.*, 2017

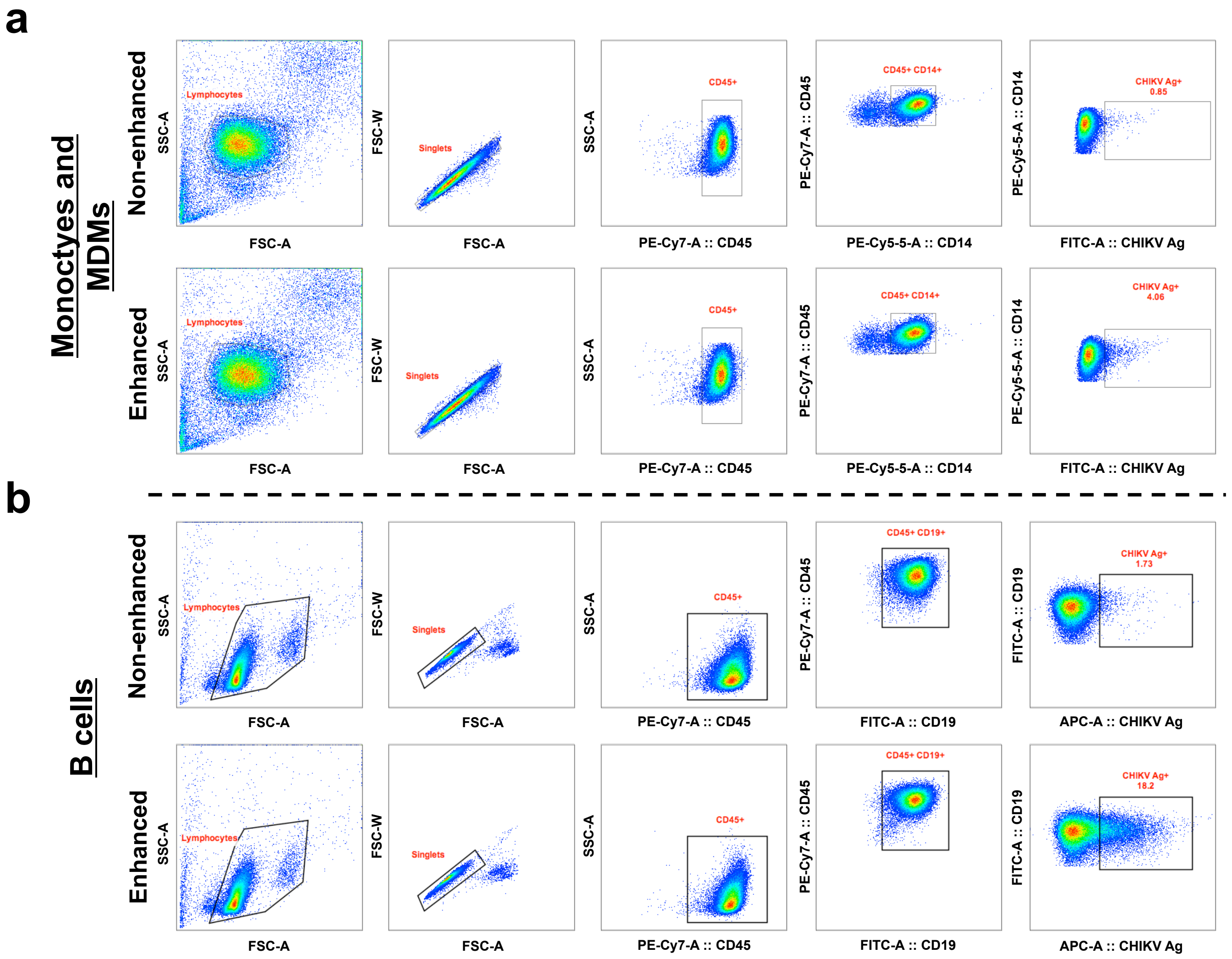

Figure S5: Gating strategy in determining CHIKV infection in infected primary human cells. Cells were first gated based on their forward and side scatter. Subsequently doublets were excluded from the analysis. Isolated primary cells were then gated based on their specific markers before percentage of CHIKV positive cells were determined from these cells. Illustration is shown using isolated primary human (**a**) monocytes and (**b**) B cells from one experiment. Strategy used in (**a**) was also applied for human monocytes-derived macrophages. Strategy described was used for all replicates of related experiments.

Fig S5_Lum *et al.*, 2017

Table S1. Sequences of primers used for gene expression studies

Genes	Primers sequences[a]	
IL-6	Forward	TTCCATCCAGTTGCCTTCTTG
	Reverse	TTGGGAGTGGTATCCTCTGTGA
iNOS	Forward	CTGCTCACTCAGCCAGGCCCTCACCTACTT
	Reverse	TGATCCCGGGAGGAGCTGATGGAGTAGAAC
SOCS3	Forward	ATGGTCACCCACAGCAAGTTT
	Reverse	TCCAGTAGAATCCGCTCTCCT
IFNα	Forward	GTGACAGGGAACTTAGGGAGC
	Reverse	AGAGTGGGGTGTCTGCCTTA
IFNβ	Forward	AACTTTGACATCCCTGAGGAGATTAAGCAG
	Reverse	GACTATGGTCCAGGCACAGTGACTGTACTC
IFNγ	Forward	ATCTGGAGGAACTGGCAAAA
	Reverse	TGAGCTCATTGAATGCTTGG
IRF3	Forward	GGGGAGCCTCTTGACTGAAAACCGTGGA
	Reverse	TAACCACCAGCCTAGACGCAGTCGACAGCA
IRF9	Forward	GCCGAGTGGTGGGTAAGAC
	Reverse	GCAAAGGCGCTGAACAAAGAG
STAT1	Forward	GCTGGGCGTCTATCCTGTGGT
	Reverse	GCTCAGCTGGTCTGCGTTCA
STAT2	Forward	TCCTGCCAATGGACGTTCG
	Reverse	GTCCCACTGGTTCAGTTGGT
STAT3	Forward	CAATACCATTGACCTGCCGAT
	Reverse	GAGCGACTCAAACTGCCCT
STAT5	Forward	CGATGCCCTTCACCAGATG
	Reverse	AGCTGGGTGGCCTTAATGTTC
STAT6	Forward	CGCTGATAAGCCGTCTGGAT
	Reverse	TGTGTCTTCAGAACCTGCGG
Viperin	Forward	CTTCAACGTGGACGAAGACA
	Reverse	GACGCTCCAAGAATGTTTCA
ISG15	Forward	TGGGACCTAAAGGTGAAGATGCTG
	Reverse	TCAGGCGCAAATGCTTGATCAC
DEF14	Forward	TCCAGGGGACGCATTCCTA
	Reverse	ACCGCTATTAGAACATCGACCTA
mIL-10	Forward	TGGCCTTGTAGACACCTTGG
	Reverse	AGCTGAAGACCCTCAGGATG
GAPDH	Forward	TCGTCCCGTAGACAAAATGG
	Reverse	TTGAGGTCAATGAAGGGGTC

[a] Primers sequences shown are of mouse origin
